# Slim-panel holographic video display

**DOI:** 10.1038/s41467-020-19298-4

**Published:** 2020-11-10

**Authors:** Jungkwuen An, Kanghee Won, Young Kim, Jong-Young Hong, Hojung Kim, Yongkyu Kim, Hoon Song, Chilsung Choi, Yunhee Kim, Juwon Seo, Alexander Morozov, Hyunsik Park, Sunghoon Hong, Sungwoo Hwang, Kichul Kim, Hong-Seok Lee

**Affiliations:** 1 Samsung Advanced Institute of Technology, Samsung Electronics, Suwon, Gyeonggi-do South Korea; 2 Optic Research Group, SAIT-Russia, SRR, 12, Dvintsev street, Moscow, Russia; 3 University of Seoul, 163 Seoulsiripdaero, Dongdaemun-gu, Seoul, South Korea

**Keywords:** Electrical and electronic engineering, Displays

## Abstract

Since its discovery almost 70 years ago, the hologram has been considered to reproduce the most realistic three dimensional images without visual side effects. Holographic video has been extensively researched for commercialization, since Benton et al. at MIT Media Lab developed the first holographic video systems in 1990. However, commercially available holographic video displays have not been introduced yet for several reasons: narrow viewing angle, bulky optics and heavy computing power. Here we present an interactive slim-panel holographic video display using a steering-backlight unit and a holographic video processor to solve the above issues. The steering-backlight unit enables to expand the viewing angle by 30 times and its diffractive waveguide architecture makes a slim display form-factor. The holographic video processor computes high quality holograms in real-time on a single-chip. We suggest that the slim-panel holographic display can provide realistic three-dimensional video in office and household environments.

## Abstract (web summary)

Holographic displays that are both compact and produce realistic holograms without eyestrain are still difficult to realize. Here the authors implement a steering-backlight unit and a holographic video processor to produce a realistic holographic display in a slim panel.

## Introduction

**Fig. 1:**
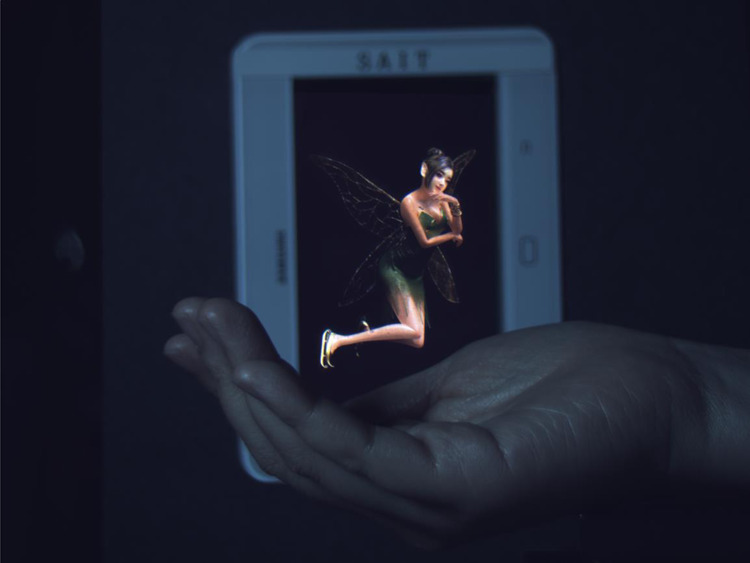
A photo of a holographic image taken with a real hand. The holographic image and the human hand are at the same distance from the camera. It provides natural depth perception and prompts a viewer to focus on the object itself, not on the screen. The bulk-optic backlight unit is used to demonstrate the ultimate quality of the holographic image. The movie clip is available in Supplementary Movie 1.

A holographic display uses light diffraction to create three-dimensional (3D) images in space^1^. When real objects and holographic images are located in the same space, they can be perceived without inhomogeneity. Figure 1 shows how the holographic image can be shown with a real human hand. Both the fairy image and the hand are 0.3 m away from the screen. With a holographic display, one can see the fairy image and the hand clearly focused without having any uncomfortable feeling. On the contrary, conventional stereoscopic 3D images using only binocular parallax and vergence are recognised as images at the screen^2,3^. One cannot clearly see the fairy image and the real hand at the same time, and will experience visual fatigue caused by an accommodation–vergence conflict^2^. Therefore, holographic displays are essential parts of future video systems including interactive 3D user interfaces.

Because of its advantage in 3D image reproduction, the technology for static holograms is quickly developed to a high standard by using hologram recording materials such as silver halide and photopolymer^4^. Recently, nanophotonics^5^ and metasurfaces^6^ are also used to reconstruct static holograms. However, those holographic media are non-updatable or have a limited updating frequency^7^, causing a fundamental limitation for generating dynamic holograms. By using a spatial light modulator (SLM), which directly modulates the wavefront of light, it is possible to update holograms at video rate. Many researches have demonstrated holographic video systems so far, including anisotropic leaky mode^8^ and eye tracking^9^. The recent study on a holographic display was reported, including a full-colour 3D computer-generated hologram (CGH) calculated in real-time, and a focus-adjustable reconstructed image^10^. However, it has been demonstrated that vertical parallax only holograms using a bulky optical system. It is expected to require quite an amount of time to reach the mobile holographic video for practical applications.

To build a mobile holographic video display, the following barriers need to be overcome. First of all, there is the limitation of the space-bandwidth product (SBP), which determines both the size of holographic image and the viewing angle. The static holographic media can produce large holographic images with a large viewing angle, because the information of hologram is recorded in the sub-wavelength density and it can be recorded on the large size film. However, SBP is limited by the pixel size and the number of pixels when an SLM is used as a dynamic holographic medium. The SBP of the currently available SLM is generally a few hundred times less than the SBP of the static holographic media. It means that only a small size or a narrow viewing angle dynamic hologram can be realised. Second, to generate a large coherent backlight, complicated optical components and a considerable space is required for the manipulation of light. It is difficult to realise a holographic video display as slim as flat-panel displays commercialised nowadays. Last, the calculation of hologram in real-time typically requires huge computational cost, and the amount of computation increases as the SBP increases. Several studies have been carried out to optimise algorithms and to increase computation speed, but they still require clustered processors or high-performance parallel processing systems to calculate high-quality hologram at video frame rate^11–16^.

In this research, a real-time interactive slim-panel holographic video display is demonstrated for the first time. It resolves all the above issues of low SBP, bulky optical system, and enormous computational cost. To increase effective SBP (Supplementary Note 1), a steering-backlight unit (S-BLU), which consists of a coherent-BLU (C-BLU) and a beam deflector (BD) is introduced. The effective SBP is increased by 30 times compared to the original value by using the S-BLU. It is the highest SBP ever achieved for a real-time holographic video system using an SLM. All the optical components are designed and fabricated as a slim structure. To generate 4-K (3840 × 2160) ultra-high-definition (UHD) pixel holograms in real-time, a holographic video processor is implemented on a single-chip field programmable gate array (FPGA).

## Results

### Beyond SBP

In holographic display, SBP can be represented by the equation, *W* × *θ* = *λ* × *N*, where *W* is the image size, *θ* is the viewing angle, *λ* is the wavelength, and *N* is the number of pixels. For a given number of controllable pixels, the maximum size of holographic image and the viewing angle are always trade-off relation. For example, an SLM with full high-definition (FHD) resolution can provide the viewing angle of 0.25° in 10-in. display or 30° in 0.1-in. display. For satisfying both large size and large viewing angle, the SBP should be expanded by increasing the number of pixels *N*. There are studies to increase the number of pixels *N* by tiling many SLMs^17^ or by increasing the resolution of a single SLM^18^. To achieve the 30° viewing angle in 10-in., 221-K horizontal resolution is required, which is ~100 times higher resolution than the FHD resolution. Apart from the difficulty in fabricating such an SLM, the computation of the CGH in real-time may not be feasible, because the total number of pixels increases ~13,000 times.

**Fig. 2:**
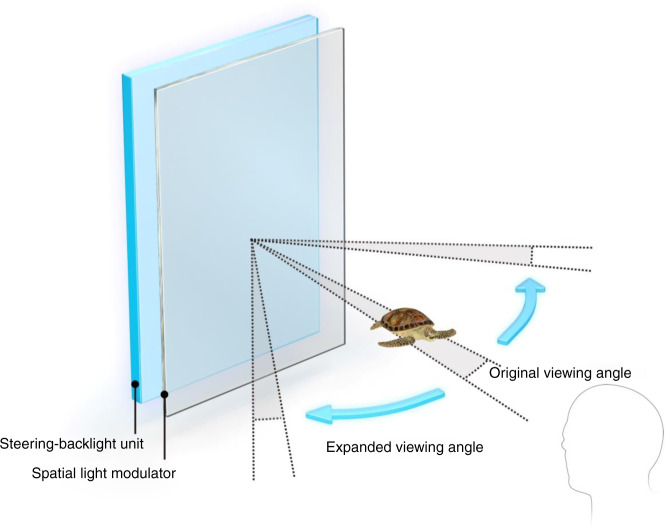
The schematic diagram of expanding effective SBP by using steering-backlight unit. In addition to the original viewing angle of holograms provided by the spatial light modulator, the steering-backlight unit effectively enhances the overall viewing angle.

In this research, we expand the effective SBP without increasing the number of pixels of the SLM. To overcome the small viewing angle of a large size SLM, we introduce S-BLU, which is able to tilt the angle of the backlight for reconstructing holograms as shown in Fig. 2. Since the S-BLU steers the backlight and can deliver the holographic image to a desired direction, the viewing angle can be effectively expanded for an observer by the amount of maximum steering angle of S-BLU.

The BD, one of the key elements of S-BLU, plays the role of steering a beam. The BD consists of transmission phase arrays, which can provide the linear phase profile for tilting the transmission angle. By using the BD, an extra enhancement factor is added to the original SBP equation *W* × *θ* = *λ* × *N*(1 + *p*_SLM_/*p*_BD_). The enhancement factor proportionally increases with the ratio of pixel pitches between SLM and BD. In our holographic video system, the pixel pitch of the BD, *p*_BD_, is designed as 2 µm while the pixel pitch of the SLM, *p*_SLM_ is 58 µm. Then, the effective SBP is increased ~30 times without increasing the number of pixels in the SLM. Since the *p*_BD_ can be reduced further independent to the SLM, using a BD is more scalable mean to expand SBP than directly increasing the number of pixels of the SLM.

### S-BLU architecture and holographic video processor

**Fig. 3:**
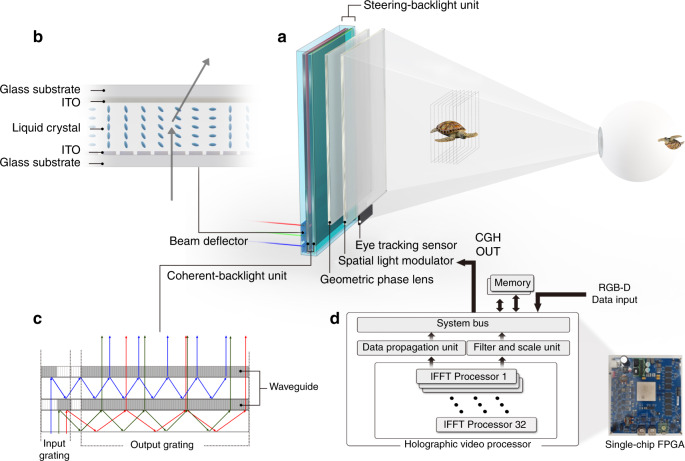
Schematics of the optical architecture with key components and the holographic video processor. **a** Optical architecture consists of beam deflectors, coherent-backlight units, a geometric phase lens and a spatial light modulator. **b** Principle of the beam deflector, which steers the transmitted lights optically like a prism: the vertical and horizontal phase arrays steer light up to ~15° with the angular resolution of 0.02° at the wavelength of 520 nm. **c** Configuration of the coherent-backlight unit using waveguide: the first waveguide for both red and green light and the second waveguide for blue light are stacked together to increase the overall efficiency. **d** The holographic video processor is implemented on a single-chip FPGA.

The S-BLU is the key component for expanding the viewing angle of holographic display. In Fig. 3a, the S-BLU consists of the BD (Supplementary Note 2) and the C-BLU (Supplementary Note 3). The BD steers light^19^ and the C-BLU^20^ forms the uniform wavefront incident from the BD as shown in Fig. 3b, c. Three sets of BDs have been used for each R/G/B colour. Each BD set consists of two pairs of linear phase arrays based on liquid crystals for both eyes. Each pair can steer light along the horizontal and the vertical directions.

In a typical beam steering, the maximum steering angle is reduced as the area of the light source increases due to the etendue. In our holographic video system, the etendue issue has been solved by using the waveguide structure of the C-BLU. Once the beam size of 14 mm × 140 mm is steered as wanted by the BDs, it is enlarged to 140 mm × 230 mm by duplicating inside of the waveguide without reduction of the maximum steered angle.

One disadvantage of the small viewing angle is a long viewing distance where the diffracted lights from all pixels start to overlap. Thus, a lens is necessary to reduce the viewing distance by gathering those diffracted lights together. The minimum viewing distance is, *D* = 0.5 × *L*/tan(*θ*/2), where *L* is the horizontal length of the display and *θ* is the viewing angle. The minimum viewing distance is ~25 m when *L* is 22 cm (equivalent to 10-in.) and *θ* is 0.25°. By using a lens of 1 m focal length, the viewing distance is reduced by 25 times. However, a conventional bulk lens is not appropriate for the slim-panel holographic video display in terms of its thickness and weight. The geometric phase lens (GPLs), which exploits the spatial anisotropy^21^, is implemented as a field lens. The diffraction efficiency of a GPL can reach 98% over the entire visible wavelength. The overall thickness of optical components including the GPL is 1 cm.

For the real-time interactive holographic video display, the 3D image should be updated for the position of the viewer’s eyes which is detected by the eye-tracking sensor (Supplementary Note 4). An application software, such as a graphic rendering software, or a 3D camera generate 3D images that should be projected on the viewer’s retina plane. The 3D image is provided to the holographic video processor in red, green, blue and depth (RGB-D) format. From the image on the retina plane, the optical field distribution on the eye lens plane is computed. Then, from the optical field distributions on the eye lens, the holographic patterns that should be applied to the SLM are computed. For these steps, a layer-based method is used and two-dimensional inverse fast Fourier transforms (2D IFFTs) are heavily used^22,23^. Layer-based method can generate high-quality holograms for real scenes. However, layer-based method using FFTs has been considered unsuitable for CGH hardware accelerators since FFT processors were difficult to build in large scale^15^. We built an IFFT-based holographic video processor (Supplementary Note 5) using a highly parallel architecture and several hardware-reducing techniques. Two holographic images are computed at the same time: one for the right eye and the other for the left eye. Later, they are combined into a single hologram^22,24^.

The holographic video processor consists of three major parts: data propagation unit (DPU), filter and scale unit (FSU), and 32 IFFT processors. The IFFT processors perform 2D IFFT computations required for the calculations of the optical field distribution on the eye lens and the hologram patterns for the SLM. The 32 IFFT processors can compute 32 1-D IFFTs at the same time. The 2D IFFTs are computed with the row–column method. The DPU prepares the data needed for the IFFT computations from various sources including the input RGB-D data. The FSU performs several filtering operations and miscellaneous functions. The FSU also performs pixel encoding, which converts a complex number into an integer, which is provided to the SLM.

The holographic video processor uses fixed-point number representations for all data processing tasks including multiplications in the IFFT. The number of bits used to represent a number is kept at the lowest level. For example, when the Fourier coefficients are stored in the memory, only 32 bits are used to represent a complex number. The holographic video processor is built with a single-chip FPGA. The single-chip FPGA holographic video processor generates binocular holographic colour images with 3840 × 2160 pixels at the speed of 30 frames per second (fps).

As the system bus, we used Advanced Microcontroller Bus Architecture Advanced Extensible Interface 4 (AMBA AXI4), which is widely used in smartphone application processors. This makes it easy to embed the holographic video processor into a smartphone application processor.

### Device implementation

**Fig. 4:**
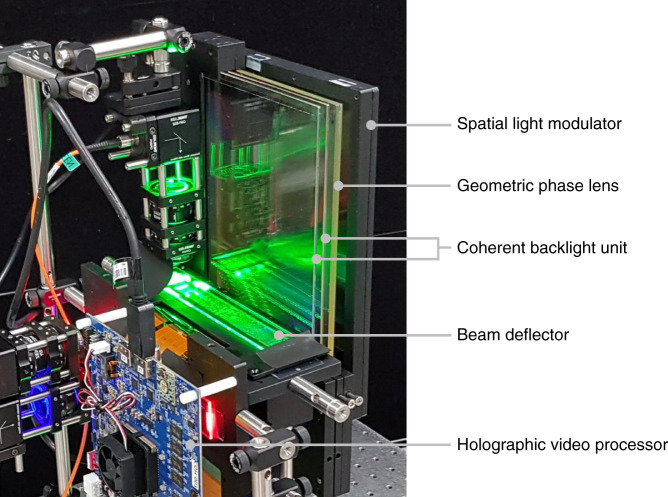
Slim-panel holographic video display. The prototype includes optical components (beam deflectors, coherent-backlight units, and a geometric phase lens), a holographic video processor board, a spatial light modulator, power connectors, and other miscellaneous components. The thickness of the display parts is 1 cm, including coherent-backlight units, a geometric phase lens, and a spatial light modulator.

The actual system implementation of the slim-panel holographic video display is shown in Fig. 4. Three colour laser diodes are used as coherent light sources. These laser lights are summed as they pass through BDs and C-BLUs, and the resulting coherent white light is led through a GPL with a focal length of 1 m onto the SLM. A 10.1-in. UHD commercial liquid crystal display (LCD) is used as the amplitude-only SLM.

Two experiments are conducted to validate the dynamic colour holographic display. First, the accommodation effect is checked by placing voxels in the air with different depths. Second, a full-screen colour holographic video is played. Each scene consists of 8 million voxels and it is refreshed at 30 fps. The CGH is calculated by the single-chip holographic video processor from a 3D scenery source rendered in the Unity platform.

**Fig. 5:**
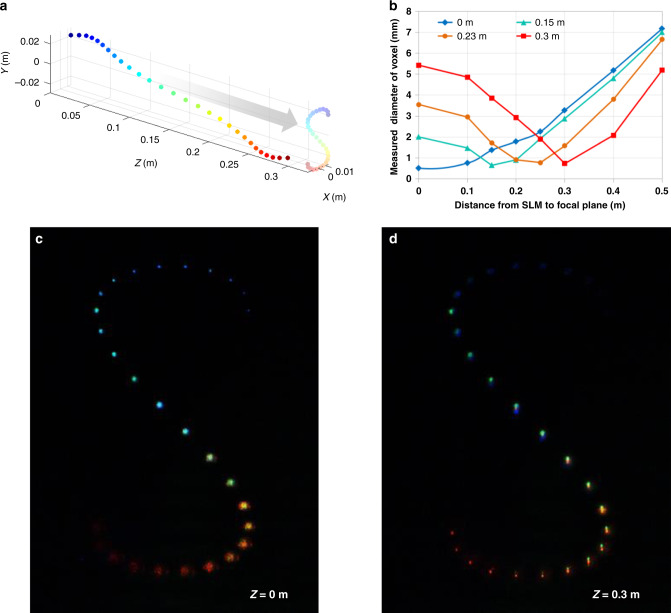
Measuring the accommodation effect. **a** The coloured voxels are placed along *Z*-axis with various *X*–*Y* positions where whole voxels can be seen as letter ‘S’. **b** Measured diameters of each voxels are plotted for different focal planes. **c**, **d** The holographic images are taken at the two focal planes of *Z* = 0 and 0.3 m, respectively. The diameter of blue voxel is the minimum at *Z* = 0, while the diameter of red voxel is the minimum at *Z* = 0.3 m, which provides the correct accommodation effect.

As shown in Fig. 5a, the voxels are placed along *Z*-axis with different *X*–*Y* positions, so that overall shape forms the letter S, when they are seen at *Z* = 1 m position. By changing the focal plane of the camera at the distance of 0 and 0.3 m, different images are captured as shown in Fig. 5c, d. Figure 5b shows the diameter of voxels along with various focal planes of camera. The distance of the minimum diameter of each voxel corresponds to the depth of its desired position. This proves that the holographic display provides the correct accommodation effect.

As the depth of voxel increases, a minimum diameter of voxel slightly increases due to the finite spatial coherence of the backlight. The contrast of the letter S is ~200. Unlike the other SBP improving method^25^, the contrast ratio does not change regardless of the number of voxels in our case.

**Fig. 6:**
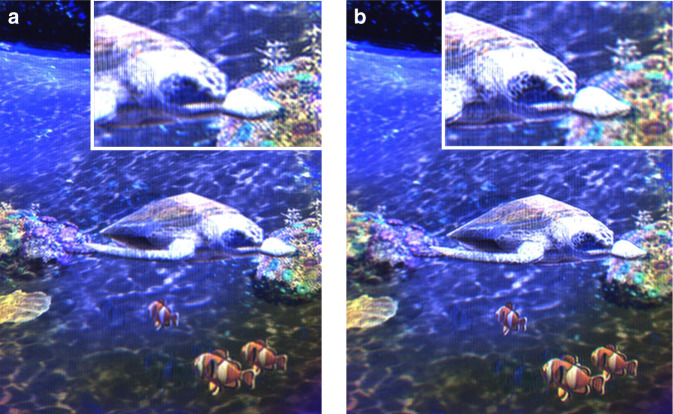
Full colour real-time holographic movie. Photos are taken from a holographic video display when it is focused. **a** 0.1 m behind the display and **b** 0.35 m in front of the display: for the comparison between the images, expanded images are shown in the upper right corners respectively. The movie clip is available in Supplementary Movie 2, where interactive holographic colour images are shown at 30 fps.

Figure 6 shows still shots taken from the dynamic holographic video display in Supplementary Movie 2. The scene is about a turtle swimming under the sea. The corals are placed 0.1 m behind the display and the hill is far behind the display panel, while the turtle and other fish move all around the space. Since the depths of all objects are different, the sharpness of holographic objects changes as the camera focus changes. The top-right inset in Fig. 6a shows that the coral is clearer than the turtle, while the inset in Fig. 6b shows that the face of the turtle becomes clear when the camera focuses it. It demonstrates the unique feature of hologram providing the correct accommodation effect as real objects do, which has not been realised by conventional 3D displays. The turtle is manually controlled along any direction by using a keypad. It proves the dynamic holographic image is updated in real-time using the holographic video processor.

## Discussion

In this paper, a novel slim-panel holographic video display is proposed mainly on a new S-BLU architecture and a single-chip real-time holographic video processor. The S-BLU expands the effective SBP by 30 times, while the holographic video processor calculates high-quality CGH at 30 fps. The overall system thickness is <10 cm. The thickness of the display parts is 1 cm. Based on the combination of these, we demonstrated the world’s first slim full-colour holographic video display using 10.1-in. conventional UHD LCD. Research is in progress to scale down the volume of the system suitable for mobile phones. The holographic video processor is also designed to be embedded in a mobile application processor. The proposed system will accelerate the adoption of the holographic video for mobile devices.

## Supplementary information

Supplementary Information

Description of Additional Supplementary Files

Supplementary Video 1

Supplementary Video 2

## Data Availability

All relevant data that support the findings of this work are available from the corresponding author upon reasonable request. Source data are provided with this paper.

## Source data

Source Data

## References

[1] Gabor D (1973). Holography, 1948–1971 [Nobel Lecture, December 13, 1971]. Sov. Phys. Uspekhi.

[2] Kim J, Kane D, Banks SM (2014). The rate of change of vergence-accommodation conflict affects visual discomfort. Vis. Res..

[3] Shibata T, Kim J, Hoffman DM, Banks MS (2011). The zone of comfort: Predicting visual discomfort with stereo displays. J. Vis..

[4] Colburn WS, Haines KA (1971). Volume hologram formation in photopolymer materials. Appl. Opt..

[5] Sun J, Timurdogan E, Yaacobi A, Hosseini ES, Watts MR (2013). Large-scale nanophotonic phased array. Nature.

[6] Ni X, Kildishev AV, Shalaev VM (2013). Metasurface holograms for visible light. Nat. Commun..

[7] Blanche P-A (2010). Holographic three-dimensional telepresence using large-area photorefractive polymer. Nature.

[8] Smalley D, Smithwick Q, Bove V, Barabas J, Jolly S (2013). Anisotropic leaky-mode modulator for holographic video displays. Nature.

[9] Häussler, R., Schwerdtner, A. & Leister, N. Large holographic displays as an alternative to stereoscopic displays. *Proc. SPIE* **6803**, 1–9 (2008).

[10] Häussler R, Gristal Y, Zschau E, Missach R, Sahm H, Stock M, Stolle H (2017). Large real-time holographic 3D displays:enabling components and results. Appl. Opt..

[11] Shimobaba T, Kakue T, Ito T (2016). Review of fast algorithms and hardware implementations on computer holography. IEEE Trans. Ind. Inf..

[12] Tsang PWM, Poon TC (2016). Review on the state-of-the-art technologies for acquisition and display of digital holograms. IEEE Trans. Ind. Inf..

[13] Chen JS, Chu D (2016). Realization of real-time interactive 3D image holographic display [Invited]. Appl. Opt..

[14] Niwase H (2016). Real-time electroholography using a multiple-graphics processing unit cluster system with a single spatial light modulator and the InfiniBand network. Opt. Eng..

[15] Sugie T (2018). High-performance parallel computing for next-generation holographic imaging. Nat. Electron..

[16] Corda R, Giusto D, Liotta A, Song W, Perra C (2019). Recent advances in the processing and rendering algorithms for computer-generated holography. Electronics.

[17] Sasaki H (2014). Large size three-dimensional video by electronic holography using multiple spatial light modulators. Sci. Rep..

[18] Lee HS (2018). Large-area ultra-high density 5.36” 10Kx6K 2250 ppi display. SID Dig..

[19] Kim Y (2018). Electrically tunable transmission-type beam deflector using liquid crystal with high angular resolution. Appl. Opt..

[20] Choi C (2015). Ultra-slim coherent backlight unit for mobile holographic display. Proc. SPIE.

[21] Kim J, Li Y, Miskiewicz MN, Oh C, Kudenov MW, Escuti MJ (2015). Fabrication of ideal geometric-phase holograms with arbitrary wavefronts. Optica.

[22] Kim H (2018). A single-chip FPGA holographic video processor. IEEE Trans. Ind. Electron..

[23] Im D (2014). Phase-regularized polygon computer-generated holograms. Opt. Lett..

[24] An J (2015). Binocular holographic display with pupil space division method. SID Symp. Dig. Tech. Pap..

[25] Yu H, Lee K, Park J, Park Y (2017). Ultrahigh-definition dynamic 3D holographic display by active control of volume speckle fields. Nat. Photon..

