# Supplementary material for: Slim-panel holographic video display
Source: Nat Commun. 2020 Nov 10;11:5568. doi: 10.1038/s41467-020-19298-4 (PMC7655945; doi:10.1038/s41467-020-19298-4)
Supplement: Supplementary file 1 — Supplementary Information [file 41467_2020_19298_MOESM1_ESM.pdf]

Supplementary Information

for Slim-panel holographic video display

An and Won et al.

Supplementary Information

Supplementary Note 1: Effective space bandwidth product

The space bandwidth product (SBP) can be generally expressed as the product of the image size and the viewing angle. In the concept of effective SBP, the viewing angle is effectively expanded by steering the entire hologram to the angle of interest. To do so, an additional optical element is required to dynamically control the optical axis of a beam. The beam deflector (BD) which consists of optical phase arrays is able to steer the beam depending on its phase profile. Like a spatial light modulator (SLM), the beam deflector has a maximum diffraction angle determined by the pitch of cells. In the presence of the beam deflector, the spatial bandwidth product can be written as below.

$$W \cdot \theta = (N \cdot p_{SLM}) \cdot \theta_{eff} \qquad \text{(eq. 1)}$$

W is the size of hologram, N is the number of pixels in a SLM, p_{SLM} is the pixel pitch of the SLM and θ_{eff} is the effective viewing angle determined by both SLM and BD. By combining two diffraction grating equations, one can derive the θ_{eff} as below.

$$\sin\psi - \sin\theta_{in} = \lambda/p_{BD}$$

$$\sin\theta_{eff} - \sin\psi = \lambda/p_{SLM}$$

$$\sin\theta_{eff} = \lambda/p_{SLM} + \lambda/p_{BD}$$

θ_{in} is the incident angle to the BD, which is zero for normal incident. ψ is the deflected angle by BD and also is the incident angle to the SLM. p_{BD} is the cell pitch of the BD. λ is the wavelength of light.

In paraxial approximation, $\sin\theta_{eff} \sim \theta_{eff}$

$$\theta_{eff} = \lambda/p_{SLM} + \lambda/p_{BD} \qquad \text{(eq. 2)}$$

By substituting the equation 2 into the equation 1, the effective SBP is obtained as below.

$$W \cdot \theta = (N \cdot p_{SLM}) \cdot (\lambda/p_{SLM} + \lambda/p_{BD})$$

$$W \cdot \theta = \lambda \cdot N \cdot \left(1 + \frac{p_{SLM}}{p_{BD}}\right)$$

where, $\left(1 + \frac{p_{SLM}}{p_{BD}}\right)$ is the enhancement factor from the original SBP value.

Supplementary Note 2: Beam deflector

Due to the anisotropic optical characteristic of liquid crystal (LC), the effective refractive index is changed when an external voltage is applied across a LC cell. The linear phase difference between neighbouring electrodes deflects the transmitted lights optically like a prism.

We designed the beam deflector system so that the refractive index difference, Δn, and the cell gap, d, can modulate the phase larger than 2π for satisfying the phase matching condition. The choice of the liquid crystal plays an important role in the electro-optical performance and diffraction efficiency. The liquid crystal mixture with a large refractive index ($\Delta n = 0.33$) is exploited for our beam deflector and the additional properties are as follows: Extraordinary refractive index (n_e) is 1.869, ordinary refractive index (n_o) is 1.539 and dielectric anisotropy (Δε) is 15.1. Due to the high birefringence of liquid crystal, the full 2π phase modulation is realised with a small cell-gap of 2.5 μm at the full spectrum of visible light. The rubbing method is used to align the LC layer homogeneously and the rubbing directions for upper and lower substrates are kept parallel. The zero-twist of LC molecules, known as electrically controlled birefringence mode, modulates phase rather than amplitude.

The maximum and the minimum steering angles are determined by the pitch of electrodes and the channel number. The maximum steering angle is $\theta_{max} = \arcsin(\lambda/2p)$ and the minimum angle $\theta_{min} = \arcsin(\lambda/np)$, where λ is the wavelength of the light, p is the pitch, and n is the number of channel. A driving module for continuous beam steering is developed and the total channel number is 720. To extend a steering angle for a wide viewing angle, a small pitch is required for the beam deflector. A reduction stepper, one of the lithography processes, was used to increase the resolution of a mask. The stepper with 5:1 reduction magnification delivers critical resolution down to 0.5 μm for realising a 2 μm pitch of indium tin oxide grating pattern (1.5 μm electrode, 0.5 μm spacing) on 0.7 mm thickness glass substrate. The maximum steering angle is

±7.47° with an angular resolution of 0.02° at the wavelength of 520 nm. The active area size to steer the incident beam is 140 mm × 14 mm and the total size including peripheral area is 175 mm × 50 mm.

Supplementary Note 3: Coherent-backlight unit

The coherent-backlight unit (C-BLU) expands the aperture of the line shaped light steered by the beam deflector. Even though the aperture of the light is expanded, the collimation angle and the steered angle should be preserved. If we use a magnifying lens which conserves optical etendue while the aperture is expanding about 17 times, the steering angle will be decreased and a huge form factor will be unavoidable. The proposed C-BLU which can handle full colour consists of two separate waveguides, one for red/green and the other for blue. The size of waveguide's in-coupling grating is 28 mm × 140 mm, which is doubled of input beam size for coupling tolerance. The size of the out-coupling grating is 140 mm × 230 mm which is slightly larger than the size of the SLM aperture.

The pitches of the in- and out-coupling grating are determined to cover the beam deflection angle of ±6°. For the maximising diffraction efficiency, each colour requires its own optimal depth of the grating. However, two types of C-BLU for the red (638 nm) / green (520 nm) and for the blue (460 nm) are selected to reduce the total thickness. The depth of the out-coupling grating is 80 nm for three colours, however, the depths of the in-coupling gratings are 120 nm and 130 nm for the blue and the red/green, respectively. The out-coupling grating is divided into small regions along the direction of light propagation. The diffraction efficiency of each region is optimised for maximising uniformity and efficiency using ray-tracing simulation with rigorous coupled-wave analysis grating model.

Due to the overlapping among out-coupling beams, dark and bright stripe patterns are shown when the beam width and the period of output coupling mismatches. To remove the stripe noise, the beam splitter coating is introduced at the interface between two substrates having different thickness. The number of beams is exponentially increased in the waveguide because the beam is split into two beams whenever the beam hits the coating. And the intensity fluctuation due to overlap will be decreased to $1/2^n$, where n is the number hits the layer.

The fabrication process consists of four steps. The first step will be the fabrication of the Si master. The Si master for the nano-imprinting process is patterned using ArF scanner to obtain sub-wavelength sized patterns. The pitches of the designed grating for the red/green and the blue are 165-335 nm and 125-245 nm, respectively. The patterned Si substrate is developed and etched. In the second step, the fabricated Si master is replicated on the PET film as a film stamp. In the third step, using the film stamp, the grating is imprinted on the BLU glass substrate of 2.2 mm thickness. Finally, to maximise efficiency, the silver layer of about 50 nm thicknesses is deposited on the in-coupling grating. To minimise the periodic dark and bright stripe patterns, the additional glass substrate of 1.5 mm thickness is attached to the backside of waveguide.

The thicknesses of the C-BLU fabricated on 280 mm × 150 mm borosilicate glass substrates for the red/green and the blue are 3.7 mm and 2.2 mm, respectively. The overall efficiency of C-BLU is 10% and the intensity uniformity is 50% adopting the uniformity compensation algorithm which adjusts the transmittance of computer generated hologram (CGH) according to the local brightness of C-BLU.

Supplementary Note 4: Holographic images at expanded viewing angles using the beam deflector

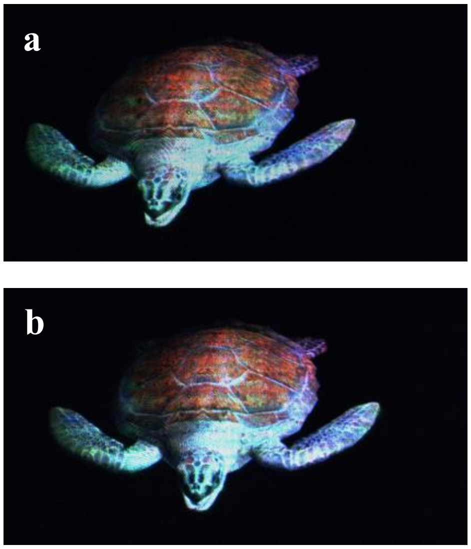

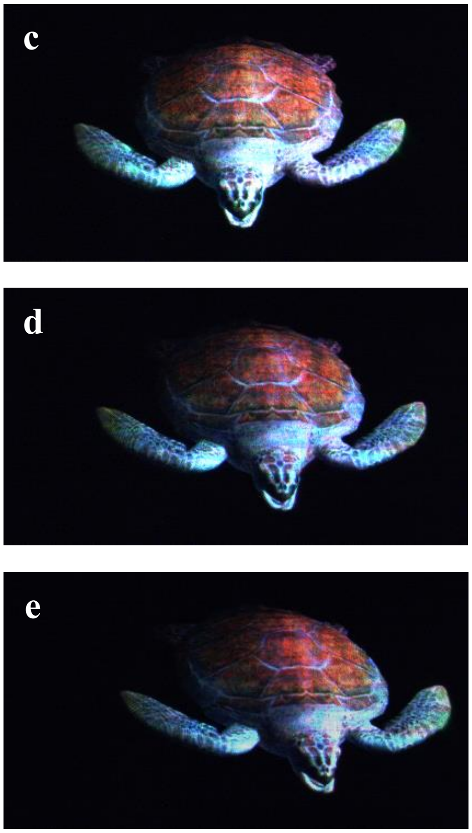

Supplementary Fig. 1 Captured holographic images from different angles. Photos are experimentally captured from a holographic video display when it is focused 200 mm in front of the display. It provides different images observed from different viewing angles **a** -6° **b** -3° **c** 0 **d** +3° **e** +6°.

The turtle holographic images in Supplementary Fig. 1 are captured at the eye positions correspond to the steering angles from -6° to +6°. The turtle is placed 200 mm in front of the panel and the hologram is updated in the SLM according to each eye position. This allows user to observe the holographic image at different side views according to the relative position with respect to the panel. The eye-tracking system cannot be used because the above images are captured using a camera rather than human eyes. For this reason, it was captured at each location by manually controlling the beam deflector.

Supplementary Note 5: Holographic video processor

The holographic display has a viewing zone of 8.8 mm × 8.8 mm. The S-BLU adjusts the position of this viewing zone to enlarge the area that the viewer can see the holographic image. When the viewer's head moves around, the direction of the light from the S-BLU is changed such that the viewer's eyes remain in the viewing zone. The position of the viewer's eyes is detected by eye-tracking sensors. To implement motion parallax, the holographic image should be updated for every new position in real-time. It also should be updated whenever there is a change in the holographic image itself. The real-time generation of the hologram is essential. Recently, a single-chip FPGA holographic video processor was introduced which can generate eight binocular colour holograms with size of 3840 × 2160 pixels per second [1]. In this work, we improved the holographic video processor such that it can generate more than 30 binocular holographic colour images with size of 3840 × 2160 pixels per second.

To achieve more than 30 frames per second, we used 32 IFFT processors. IFFT processors perform 2048 × 1024 2D IFFTs using row-column method. The IFFT processors operate in two modes: 16 processors mode and 32 processors mode. A data processing unit can read 64 bytes and write 64 bytes concurrently in a single clock through AXI4 system bus. In each clock, an IFFT processor reads and writes 4 bytes at the same time, 16 bits for real part and 16 bits for imaginary part. Only 16 IFFT processors can operate at the same time when a continuous stream of inputs and outputs is required. When 1024-point IFFTs on the columns of data are computed, IFFT processors are used in the 16 processors mode. When 2048-point IFFTs on the rows of input RGB-D data are computed, IFFT processors are used in the 32 processors mode. The concurrent use of 32 IFFT processors is possible since the input RGB-D data is made of 8-bit real numbers. 32 IFFT processors perform 2048-point 1-D IFFTs for 64 rows at the same time. When the second row-wise 2048-point 1-D IFFTs are computed, 16 IFFT processors are used for the left eye holographic image and the other 16 IFFT processors are used for the right eye holographic image at the same time. After further processing in the filter and scale unit, the holograms for each eye are combined into a single hologram. Then, the single hologram is written into the memory. It is noteworthy that the use of 32 IFFT processors reduces memory traffic considerably. The memory traffic for reading input RGB-D data is reduced to half and the memory traffic for storing data after the second 2D IFFT is eliminated. The operation frequency of all data processing units is 150 MHz. The operation

frequency of the memory interface is 300 MHz. Circuits with large critical path delay are redesigned. Techniques to reduce circuit delays are extensively used in the place and routing process. Asynchronous transfer is used for the memory traffic. There are three major memory traffics: the memory traffic for the input RGB-D data, the memory traffic for CGH output data for SLM and the memory traffic between the CGH core and the memory system. The three memory traffics are not scheduled in a fixed order. They are scheduled asynchronously independent of each other and memory traffics are overlapped. Asynchronous memory traffic helped to reduce the idling time of the CGH core to wait the input RGB-D data.

Real-time holographic videos demand an enormous amount of computation. This can lead to complex, bulky, and power-hungry hardware. Two techniques are used to reduce the complexity of the hardware and power. First, the number of operations is reduced. When the first row-wise 1-D IFFTs are computed for the input data of a given layer, the 32 IFFT processors perform 1-D IFFTs for 64 rows at the same time. This reduces the number of row-wise 1-D IFFTs by 50%. From experiments, we found that the spatial frequencies above a certain threshold frequency can be removed without a noticeable degradation of the quality of the holographic video. The Fourier coefficients above the threshold frequency are not computed. This reduces the number of column-wise 1-D IFFTs by 75%. Based on these processes, the holographic video processor can perform 2,160 2D IFFTs per second. The number of IFFT computations increases linearly as the number of layers increases. From experiments, we concluded that eight layers provide enough depth information to human eyes [1]. Secondly, we replaced high complexity operations with low complexity operations. Floating-point representations are not used in any data processing including multiplications in the IFFT. During CGH computations, pixels in a holographic image are represented with three complex numbers. These complex numbers are represented using only 32 bits each. 16 bits are used for both real part and imaginary part. This leads to a considerable reduction in the hardware for arithmetic operations. In addition to the reduction in hardware, using a small number of bits for data representation helped to reduce memory bandwidth. Since the 512 bit/clock memory bandwidth of the AXI4 bus was a serious limitation in building the holographic video processor, 32 bit/pixel was firmly abided. When the desired precision was not achieved with the 32-bit representation, temporal increase in the number of bits was allowed. However, the number of bits is reduced to 32 bits before the data is written to the memory. Complex operations

are replaced by simple operations whenever it is possible. Lookup tables (LUTs) are widely used to replace complex mathematical operations. The number of bits used for the LUTs is also kept as small as possible.

We used Xilinx Kintex UltraScale FPGA (XCKU115- FLVA1517-2-E) for the holographic video processor. We used DisplayPort 1.2 and Xilinx DisplayPort intellectual property (IP). We used two double data rate 4 (DDR4) memory modules and Xilinx memory interface generator IP. We used a 300 MHz clock for the DDR4 memory interface and a 150 MHz clock for all the other data processing units. The holographic video processor uses 258942 (39.03%) LUTs, 987 (45.67%) block random access memories (RAMs), and 665 (12.05%) digital signal processor slices in the FPGA chip. The holographic video processor performs approximately 140 Giga operations per second. It is noteworthy that the holographic video processor performs considerably less amount of computations than recent works but provides a high-quality holographic video in real-time [2, 3].

Supplementary References

[1] Kim, H. et al. A single-chip FPGA holographic video processor, IEEE Trans. Ind. Electron. 66, 2066-2073 (2018).

[2] Sugie, T. et al. High-performance parallel computing for next-generation holographic imaging. Nat. Electron. 1, 254-259 (2018).

[3] Corda, R., Giusto, D., Liotta, A., Song, W. & Perra, C. Recent Advances in the Processing and Rendering Algorithms for Computer-Generated Holography, Electronics 8, 556 (2019).
