# Supplementary material for: Slim-panel holographic video display
Source: Nat Commun. 2020 Nov 10;11:5568. doi: 10.1038/s41467-020-19298-4 (PMC7655945; doi:10.1038/s41467-020-19298-4)
Supplement: Supplementary file 2 — Description of Additional Supplementary Files [file 41467_2020_19298_MOESM2_ESM.pdf]

Description of Additional Supplementary Files

File name: Supplementary Movie 1
Description: A full colour holographic video using a bulk-optic backlight unit. Movie clip is experimentally recorded for a full colour holographic video using a bulk-optic backlight unit. It is corresponding to Fig. 1 in the main article. The holographic image, the fairy, is moving around in a range of 0 to 0.3 m distance from the panel. The movie is taken 1 m in front of the LCD panel and the camera keeps focusing on the fairy.

File name: Supplementary Movie 2
Description: A real-time interactive slim-panel holographic video. Movie clip is experimentally recorded for a real-time interactive slim-panel holographic video. It is corresponding to Fig. 6 in the main article. The movie is taken 1 m in front of the LCD panel. The movie shows images of the holographic video with real objects such as water plants. The user can interact with the turtle in real-time by using a keypad.
